# Introducing a Puppy to Existing Household Cat(s): Mixed Method Analysis

**DOI:** 10.3390/ani12182389

**Published:** 2022-09-13

**Authors:** Rachel H. Kinsman, Sara C. Owczarczak-Garstecka, Rachel A. Casey, Rosa E. P. Da Costa, Séverine Tasker, Jane K. Murray

**Affiliations:** 1 Dogs Trust, London EC1V 7RQ, UK; 2 Bristol Veterinary School, University of Bristol, Bristol BA6 8DD, UK; 3 Linnaeus Veterinary Limited, Solihull B90 4BN, UK

**Keywords:** dogs, cats, cat-dog interactions, cat-dog relationships, intraspecific relationships, behaviour, puppy behaviour, cat behaviour, dog behaviour

## Abstract

**Simple Summary:**

Cats and dogs can form friendly relationships and conflict between them can harm their welfare. This study aimed to investigate factors related to ‘only desirable’ puppy behaviour (defined as being uninterested, ignored the cat or interacted in a calm way) towards existing household cats following introduction. Owners’ perceptions of the emerging cat-dog relationships were also explored. Quantitative and qualitative analyses were used to examine survey data collected as part of an ongoing study of dog health and behaviour. Of 4678 puppies, 26.7% lived with at least one cat. At the time of survey completion, 1211 puppies had been introduced to the household cat. The most common behaviours shown towards cats by puppies were: playing (58.9%), being overexuberant or over-excited (56.6%) and chasing (48.6%). Just 7.3% of puppies showed ‘only desirable’ behaviours. Puppies were more likely to show ‘only desirable’ behaviours when introduced before 12 weeks of age, gradually, and when they lived in a household with other dogs(s). Two styles of introductions were identified—owner-led and pet-led. Owners’ expectations of the cat-dog relationship differed depending on the style of introduction. Early, gradual, owner-led introductions of puppies to household cats can help them form a friendly relationship.

**Abstract:**

Although cats and dogs can live amicably, inter-species conflict can result in poor welfare. Species introduction can impact the development of the cat-dog relationship. This study aimed to identify factors associated with owner reported ‘only desirable’ puppy behaviour (defined as the puppy being uninterested, ignored the cat and/or interacted in a calm way) following introduction to existing household cats, and to explore perceptions of the emerging cat-dog relationship. Owner-reported data collected as part of a longitudinal study of canine health and behaviour were used. Of 4678 puppies, 26.7% lived with at least one cat. Of the 1211 puppies who had been introduced to the household cat at the time of survey completion, playing (58.9%), being overexuberant or over-excited (56.6%), and chasing (48.6%) were the most common behaviours displayed towards cats. ‘Only desirable’ behaviours were shown by 7.3% of puppies. Multivariable logistic regression showed early (puppies aged <12 weeks), gradual introductions and living in a multi-dog household increased the odds of ‘only desirable’ behaviours. Qualitative analysis revealed two styles of introductions—owner-led and pet-led. Owners who led introductions anticipated amicable relationships between pets, whilst owners who let pets introduce themselves did not. Early, gradual, owner-led introductions of puppies to household cats should be encouraged.

## 1. Introduction

Cats and dogs are the most common household pets worldwide. A survey of United Kingdom (UK) households reported that 7% of 3155 respondents owned both species [1]. Cats and dogs are vastly different species for many reasons including evolutionary development, social behaviour, and communication methods [2,3,4,5]. Understanding the relationships between these cohabiting species is important because their quality can impact both animals’ welfare [6] and potentially the owners’ wellbeing too [7]. Additionally, aggression towards other animals is a documented risk factor for relinquishment of both species [8,9].

An amicable cat-dog relationship can be defined as a ‘friendly, mutual bond, which is recognizable through the use of affiliative behaviours, maintaining proximity and effective, nonaggressive communication between individuals’ [10]. Thomson et al. [10] reported 82.9% of 748 participants (who owned both species) believed that their cat was comfortable in their dog’s presence, and 91.2% thought that their dog was comfortable near their cat. However, when asked how frequently their cat or dog was uncomfortable in the other’s presence, 20.5% of cats and 7.3% of dogs were reportedly uncomfortable ‘every day to once a week’. Moreover, 56.5% of cats were reported to have threatened the dog and 9.6% to have caused injury. Contrastingly, 18% of dogs were reported to have threatened the cat, and 0.9% to have caused injury. Thomson et al. [10] reported factors associated with greater owner-perceived amicability of cat-dog interactions included early age of introduction and cat housing status. Cats who spent more time indoors showed greater amicability to dogs than cats who spent more time outdoors, although this relationship may be due to cats who did not have an amicable relationship with dogs simply avoiding time-sharing [10]. 

Another study of 170 participants who owned both species reported that 66.5% of dogs and 65.5% of cats demonstrated a capacity to form amicable relationships (defined by these authors as ‘willingness to initiate proximity and/or contact’, for example, sleeping or playing together or touching each other) with the other species [5]. Two factors associated with this were the first encounter between the cohabiting animals occurring at under 6 months in cats and under 1 year in dogs and introducing the dog to an existing household cat (rather than the reverse). Early introduction between the cohabiting animals was also conducive to both species having a better understanding of the other species’ body language [5]. Thus, this research highlighted how important the cat-dog introduction may be. However, it is unclear how other aspects of the introduction, for example, the pace, household factors (such as presence of other animals or layout of the house), owners’ management of pets (such as using treats or environmental modifications) and owners’ perceptions of cat-dog relationship impact the outcome of the introduction. 

Previous research that explored rearing cats and dogs together showed that dogs tended to engage cats in play regardless of their previous experience with cats. However, play was reciprocated primarily by cats who had prior experience with dogs. Dogs who did not grow up around cats were observed to engage in more ‘rough and tumble’ play with cats, and cats who did not grow up with dogs were generally more passive and fearful when interacting with dogs [11]. Additionally, Feuerstein and Terkel [5] reported 9% of dogs and 8.5% of cats were aggressive (defined as ‘‘attacking, biting, or scratching’’) to the other species in their household, and 24.5% of dogs and 26% of cats were indifferent (defined as ‘‘avoiding contact with the other animal and/or ignoring its presence’’). However, it is possible that not all owners were able to interpret cat and dog behaviour correctly, potentially influencing these findings [5]. Whilst the previous studies examined interactions between mature cats and dogs and those reared together, to the authors’ knowledge, there has been no previous research which has examined owner-reported puppy behaviour following introduction to existing household cat(s) in great depth. This study seeks to address this.

Recommendations to aid introducing a puppy/dog to an existing household cat include looking for a breeder that also owns cats, introducing pets gradually, scent swapping, creating separate safe spaces for the dog and the cat(s), and/or keeping the dog on the lead during the initial introductions [12,13,14,15]. Offering a cat choices and control over their environment, being attentive to cat behaviour and body language, as well as limiting touch [16] have been documented to increase the frequency and duration of affiliative and positive-affect linked behaviours towards a person and a reduction in agonistic and negative-affect linked behaviours [16]. This may apply to cat-dog interactions too. Although animal welfare charities propose recommendations to aid introducing a puppy/dog to an existing household cat, little is currently known about how owners introduce dogs to household cat(s) and their experiences. Understanding how owners currently handle these introductions and identifying the issues owners faced will aid the tailoring of advice that can be given to future owners in this position. 

Given the discrepancy between the perceived acceptance and affability between cats and dogs, and frequency of amicable and aggressive behaviours shown by these pets, further research into owners’ perceptions of cat-dog interactions is needed. Increased understanding of how cohabiting cats and dogs are introduced to each other when dogs are young, and the management of early interactions, could be important in helping to create positive relationships between household cats and dogs. Therefore, the over-arching goal of this study was to improve this understanding and hopefully aid the creation of practical guidelines aimed to minimise welfare harm to cats and dogs who share a household. Consequently, this study aimed to explore:

- owner-reported puppy behaviour following introduction to existing household cat(s);
- quantitative analysis of factors associated with owner reported ‘only desirable’ puppy behaviour towards household cats following introduction; and
- qualitative analysis of owners’ approaches to introducing a puppy to a household cat and perceptions of the emerging cat-dog relationship.

## 2. Materials and Methods

### 2.1. Study Design and Participants 

The data used for this analysis were collected as part of ‘Generation Pup’—a longitudinal study of canine health and behaviour which began on 4 May 2016. The inclusion criteria for ‘Generation Pup’ were: (1) participants must be resident in the UK or the Republic of Ireland (ROI), (2) be at least 16 years old, and (3) own a puppy under 16 weeks old at time of registration (or under 21 weeks if the puppy had been through quarantine). Study methodology and recruitment methods used have been detailed elsewhere [17].

### 2.2. Data Collection and Study Size

To explore factors that could help inform recommendations for introducing puppies to existing household cats, owners were asked to select behaviours they had observed in their puppy following introduction to their household cat(s) from a predefined list. Data were gathered from three mandatory surveys which were issued to participants upon enrolment to ‘Generation Pup’ (age of the puppy varied from birth to 17 weeks old), and from three optional surveys—the ‘Settling In’, ’12 week’ and ’16 week’ surveys. The ‘Settling in’ survey was issued to owners one week after the puppy joined the household. Owners received the other two optional surveys when the puppy reached 12 weeks and 16 weeks old, respectively [17]. All surveys were self-administered, and owners could participate via online or postal surveys. All data were pseudonymised prior to analysis.

The study size was dependant on the number of owners who, at the time of data extraction (31 March 2022), had completed the three mandatory registration surveys and answered questions about introducing their puppy and existing household cat(s), which were only included in either the ‘Settling In’, ’12 week’ or ’16 week’ surveys. Although owners could register up to five puppies with ‘Generation Pup’, only one puppy per household was randomly selected for inclusion in the dataset, to eliminate any impacts of clustering at the level of the household.

### 2.3. Data Analysis

This study used a mixed methods approach to data collection, analysis, and reporting. The surveys included both close and open-ended questions, meaning that qualitative and quantitative data were collected concurrently. This mixed-methods approach helps to explain the findings of the statistical analysis and improve its validity through analytical triangulation [18]. A contiguous approach to reporting findings was taken [18]; qualitative and statistical findings are reported separately in the results section and brought together in the discussion (Section 4). The statistical and qualitative analysis is explained in detail below (Section 2.4 and Section 2.5).

### 2.4. Statistical Analysis

Descriptive statistics (frequency and percentage) were calculated for all variables. For the analysis, the puppy behaviours owners reported following introduction to the existing household cat(s) were classified into the two categories shown in Table 1. The categories were defined after consulting expert behaviourists (Certified Clinical Animal Behaviourists (CCAB) with the Association for the Study of Animal Behaviour (ASAB)). ‘Undesirable’ behaviours were those deemed less likely to result in a successful relationship between the puppy and cat(s), hence ‘undesirable’ is from the perspective of the cat. Although not all play behaviour is undesirable, some puppy playfulness may be perceived as threatening by cats. Therefore, on the advice of behaviourists, and based on the results of our statistical analysis, we categorized play behaviours as ‘undesirable’ behaviours.

For the statistical analysis, an outcome variable was created with two categories; puppies that displayed ‘only desirable’ behaviours, and puppies that displayed ‘one or more undesirable’ behaviours (with/without desirable behaviours also being displayed). Data were collected for a variety of variables of interest, such as the speed at which owners introduced their puppy to their existing household cat(s). Owners were asked to select one timeframe from a predefined list (Table 2) rather than provide free-text responses. 

Univariable and multivariable logistic regression models were used to identify which of these explanatory factors were associated with the outcome variable. 

To improve the statistical power of analysis, univariable analysis was also used to justify merging categories that had similar associations with the outcome variable, when logical. For example, this was done for the ‘Introduction speed’ variable and ‘Age of puppy when cat-dog data were collected’ variable. The age of the puppy when the behaviour data were reported was calculated using the dog’s date of birth and the date of survey completion; the age of the puppy varied from birth to 22 weeks of age. Thus, this information was summarised by the variable called ‘Age of puppy when cat-dog data were collected’ and was categorised into two groups ‘<12 weeks’ and ’12 to 22 weeks’ of age. This categorisation was based on the key socialisation period (3 to 12 weeks) [19] and the results of univariable logistic regression analyses that supported the selection of this cut off for the continuous data. 

Explanatory variables liberally associated with puppies that displayed ‘only desirable’ behaviours were identified using univariable logistic regression (*p* < 0.2). These variables were then included in the multivariable logistic regression model building process using backwards stepwise elimination. Significance for the final model was set at *p* < 0.05. The final multivariable logistic regression model was assessed using the Hosmer-Lemeshow test to evaluate the model fit quality. Additionally, the model was examined for confounding variables by re-building the model in a stepwise fashion and checking for alterations in the odd ratios of variables that were >20% [20]. Correlation matrices, variance inflation factors and tolerance were evaluated in order to check for collinearity of variables [21]. Variables with variance proportions >0.5 were considered collinear. When variables were found to be collinear, separate models were constructed containing each of the collinear variables. The log-likelihood of each model was compared to allow for the selection of the strongest model. The statistical packages IBM SPSS Statistics v26 (IBM Corp, Armonk, NY, USA) and R software version 4.1.3 (R Core Team, Vienne, Austria) were used for the data analyses.

### 2.5. Qualitative Analysis

Reflexive thematic analysis [22,23] was used to explore puppy owners’ approaches to introducing a puppy to an existing household cat and perceptions of the emerging cat-dog relationship. Free-text responses to the question “If you would like to, please give us more details about introducing your puppy to your cat(s) and how they are getting on...” asked within the ‘Settling In’, ’12 week’ and ’16 week’ surveys were analysed. The analysis was carried out by three researchers (R.E.P.D.C., R.H.K. and S.C.O.-G.). Briefly, after familiarisation with the data, initial codes were developed through inductive line-by-line coding (i.e., codes were assigned to summarise the data rather than to reflect an existing theory or pre-defined categories) [24]. Each researcher coded approximately 1/3 of the data and 15% of the text was coded by two researchers to improve rigour of the study and develop a shared understanding of the data. All available data were coded and analytical saturation (repetition of identified codes and meaning) was observed. Throughout the coding stage researchers met regularly to discuss the codes used and to produce domain summaries, i.e., summaries of the range of meaning within the data grouped by topics [22]. Later, codes were standardised between researchers and the revised coding scheme was applied to the whole dataset. Subsequently, themes were developed iteratively by exploring relationships between codes within, and between, domain summaries [22,23]. Themes were defined as patterns within the data that represent a shared meaning [25]. Qualitative data analysis was carried out in Microsoft Excel.

By taking a reflexive thematic analysis approach, critical realist methodology was followed, which assumes that the reality described during the research process is mediated by participants’ and researchers’ characteristics and context. Within this approach, the choice of words and descriptions is seen as important to understanding of individuals’ reality [25]. This approach was selected as it suits our research question and data. In realist/contextualist methodology, the codes and themes are seen as generated through the analytic process rather than discovered, therefore, researchers’ positionality and background are seen as important in shaping themes [23]. For this reason, prior and throughout the analysis the researchers discussed and recorded their assumptions and expectations regarding the data to understand how they may contribute to the analysis.

### 2.6. Ethical Approval and Consent to Participate

Ethical approval was obtained from the University of Bristol Animal Welfare Ethical Research Board (UIN/18/052), the Clinical Research Ethical Review Board at the Royal Veterinary College (URN 2017 1658-3), the Social Science Ethical Review Board at the Royal Veterinary College (URN SR2017-1116), and Dogs Trust Ethical Review Board (ERB009). Informed consent was provided by all participants in ‘Generation Pup’.

## 3. Results

This study used quantitative analysis to explore factors associated with ‘only desirable’ puppy behaviour towards household cats following introduction (Section 3.1). In order to explore this area in greater detail, qualitative analysis was used to explore owners’ approaches to introducing a puppy to a household cat and perceptions of the emerging cat-dog relationship (Section 3.2).

At the time of this study, recruitment from ‘Generation Pup’ was still ongoing, thus the analysis presented here used data from 5742 puppies registered with the study between 4 May 2016 and 31 March 2022. Following data cleaning, 788 puppies were removed due to non-completion of the surveys of interest, and 276 puppies were removed due to their owner having more than one puppy registered onto ‘Generation Pup’. Of the remaining valid sample (*n* = 4678), 26.7% of puppies (*n* = 1248) cohabited with at least one cat, whereas 73.3% of puppies (*n* = 3430) did not live with a cat.

**Figure 1:**
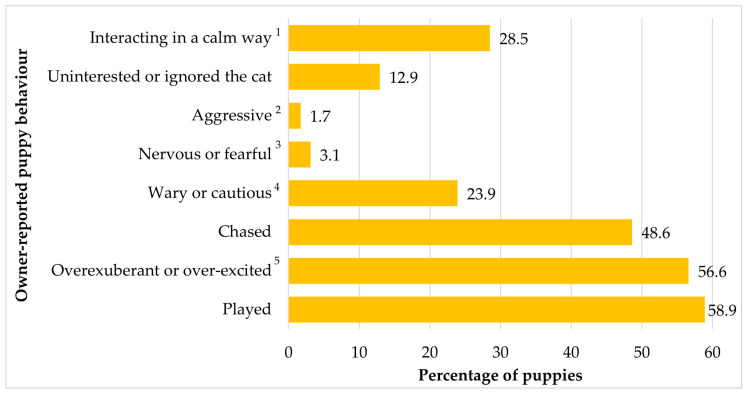
Owner-reported behaviour displayed by 1211 puppies towards existing household cat(s). ^1^ Interacting in calm way (for example sniffing or sleeping with the cat(s). ^2^ Aggressive (for example growling or snapping). ^3^ Nervous or fearful (for example moving into another room or freezing). ^4^ Wary or cautious (for example keeping a distance or cowering during interactions). ^5^ Overexuberant or over-excited (for example persistently trying to interact).

Of the 1248 puppies cohabiting with cats, 97% (*n* = 1211) puppies had been introduced to the existing household cat(s), but 37 puppies had yet to be introduced at the time of data collection. Table 3 shows the speed at which puppies were introduced to the household cat(s). Figure 1 shows the observed puppy behaviour following introduction to the existing household cat(s) as reported by the owners. Owners reported that 7.3% of puppies (*n* = 88) showed ‘only desirable’ behaviours, and 92.7% of puppies (*n* = 1123) showed ‘one or more undesirable’ behaviours (with/without desirable behaviours also being displayed).

### 3.1. Results of Statistical Analysis

Table 4 summarises the univariable logistic regression results—five of the seven variables assessed had a *p* < 0.2 and were included in the multivariable logistic regression. During the modelling, four variables were found to have a *p* value of <0.05, however, upon assessment for collinearity, two of the explanatory variables (‘Number of dogs in household’ and ‘Another dog aged ≥1 year in household’) were found to have variance proportions >0.5. The variable retained in the final model was selected by comparing the log-likelihood of a model containing the variable ‘Number of dogs in the household’ to a model containing the variable ‘Another dog aged >1 year in household’. As the first model had a higher log-likelihood, the variable ‘Number of dogs in household’ was included in the final model. Thus, the final multivariable logistic regression model identified three variables related to ‘only desirable’ behaviour from puppies following introduction to cat(s) (Table 5). These were the age of the puppy when the introduction data were collected, the number of dogs in the household and the speed of introduction between the puppy and cat(s). The Hosmer-Lemeshow test indicated acceptable model fit (*p* = 0.169).

### 3.2. Qualitative Findings

**Figure 2:**
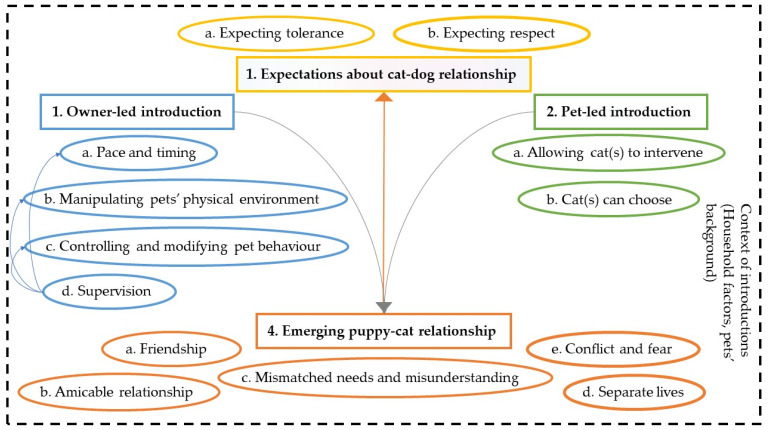
Schematic representation of themes and sub-themes that described puppy owners’ approaches to, perceptions and expectations of introducing a puppy to an existing household cat.

Qualitative data for 872 puppies were available for analysis. Five inter-related themes that described the cat-dog relationship and owners’ approaches to and expectations of introducing a puppy to a household cat(s) were generated. (See Figure 2 for schematic representation of the main themes and sub-themes). 

The theme ‘Owner-led introduction’ describes introductions where owners managed, supervised, and controlled pets’ interactions in several ways. Insights from supervision fed back into all aspects of ‘Owner-led introduction’ (for example, enabling owners to alter the pace of the interactions), and also informed ‘Owners’ expectations about puppy-cat relationship’, the second theme. By contrast, the theme ‘Pet-led introduction’ describes instances where owners allowed the cat(s) to intervene or choose when and how to interact with the puppy. The ‘Emerging puppy-cat relationships’ theme convenes different types of developing relationship between pets. Observing the ‘Emerging puppy-cat relationships’ theme also feeds into ‘Owners’ expectations about puppy-cat relationship’, which in turn was captured by two sub-themes: ‘Expecting pets to be tolerant’ and ‘Expecting dogs to be respectful’. Owners who opted for ‘Pet-led introduction’ more often expected puppies to be taught respect by the cat and were generally more accepting of ‘Mismatched needs and misunderstandings’, pets living ‘Separate lives’, or ‘Conflict and fear’ in interactions between pets. Introductions were set within the context of the cat’s and the dog’s history, the number of cats in the household and the layout of the house, all of which contributed to the relationship between pets. Table 5 shows quotes that illustrate these themes. 

Although owner and pet-led introductions are presented separately, owners often described a blended approach, where for example, they let cat(s) introduce themselves but also manipulated the pets’ environment to allow cat(s) to stay separated from dog(s) if they chose to do so. The emerging cat-dog relationships were also fluid and owners often described that although the relationship started as a mismatch of the puppy’s and the cat’s needs, over time signs of amicable relationship were visible (for example, co-sleeping or spending time in each other’s proximity; see Table 6).

## 4. Discussion

This study provides unique insights around how dog owners in the UK and ROI introduce puppies to existing household cats. More than a quarter (26.7%) of study participants reported owning at least one cat and at least one dog. This is higher than the 7% reported in a previous UK-based study by Murray et al. [1]. This variation could be due to fluctuations in the pet population and acquisition practices over time [26,27] or differences in the methodologies of the two studies and subsequently the demographics of the participants. Murray et al. [1] used a cross-sectional study design and conducted one-off telephone interviews, whereas ‘Generation Pup’ is a longitudinal study with multiple surveys requiring completion over time. Two common sources of bias in longitudinal studies are self-selection bias and under-representation of lower socioeconomic backgrounds [28]. These factors could affect how representative the participants/households in this study were compared to the general population.

Quantitative analysis of the 1211 ‘Generation Pup’ puppies living with a cat(s) showed that over a quarter were reported to interact in a calm way. Additionally, 12.9% were reportedly ‘uninterested or ignored the cat’, 3.1% were ‘nervous or fearful (for example moving to another room or freezing)’ and 1.7% were ‘aggressive (for example growling or snapping)’. Although direct comparisons are precluded due to methodological differences, as previously mentioned, Feuerstein and Terkel [5] reported 9% and 24.5% of dogs were described as aggressive and indifferent, respectively, towards the cat(s) in their household. The lower proportion of aggression and avoidance/fear-related behaviours in the ‘Generation Pup’ cohort compared to the Feuerstein and Terkel [5] study may be due to the age of the dogs studied. All ‘Generation Pup’ dogs were under 22 weeks old whereas the dogs in the previous study were aged ≥26 weeks. Younger dogs are generally more plastic in responses to new situations and contexts, whereas patterns of behaviour may become more habitual over time. Hence younger puppies may be more likely to adapt to the presence of a cat than adult dogs. With time, dogs are also likely to have experienced cats previously (for example, neighbours’ cats in the garden) and learnt responses such as chasing, whereas younger puppies introduced to a household cat are more likely to be experiencing a cat for the first time and have no pre-existing habitual response. In addition, at approximately 6 months of age (or later in larger breeds), dogs are suggested to go through a secondary sensitive period, when their sensitivity to fear-inducing stimuli, such as cats, may be increased [4,29], precipitating fear responses when dogs are exposed to cats for the first time at this age. 

Some owners reported that their puppies were playful (58.9%), overexuberant or excitable towards the cat(s) (56.6%) or chased the cat(s) (48.6%). For these behaviours, direct comparison with other studies is not possible. A puppy chasing a cat is most likely to be motivated by playfulness, as chasing and being chased are a typical part of social play for dogs [30,31]. However, chasing may also be motivated by the development of prey drive; behaviour patterns aimed at finding, pursuing, and killing prey species [32,33]. Chasing is also part of social play for kittens aged three to 16 weeks old, along with wrestling and play fighting [34]. Between around 18 and 21 weeks of age kittens switch from social play to object and predatory play, which includes capturing and handling prey (or objects), poking, batting, leaping, pouncing, grasping, and biting prey [34]. However, how play behaviour changes as cats mature is unclear as research into play motor patterns of adult cats is scarce [35]. It is likely that patterns of play related to interactions with dogs are learnt through individual experiences of living with dogs—so play behaviour of individual cats may vary considerably with prior environments. Compatibility of play behaviour between mature cats and puppies cannot be assumed, particularly if cats had no prior experience of cohabiting with a dog(s). Qualitative analysis showed that the needs of puppies and cats were often mismatched, with puppies engaging in play and chasing more than cats who rarely reciprocated play behaviour.

In the qualitative analysis, many owners interpreted puppy behaviour, such as chasing, as a game and expected cats to avoid puppies by running away, climbing out of reach, and/or not approaching areas where puppies had free range. Although these behaviours could be indicative of play, they may also be motivated by the dog’s predatory instinct. This suggests that owners may find it difficult to distinguish between play and predatory behaviour. Alternatively, owners may be able to differentiate between these two behaviours but struggle to intervene, permitting puppies to chase cats. Several owners also expressed a belief that cats do not play with dogs. Owners who did not intervene in cat-dog interactions and who opted for a pet-led introduction also expected cats to show some aggressive behaviours to the puppy and saw these behaviours as ‘necessary’ to teach the puppy to respect the cat. Owners who led on cat-dog introductions employed strategies that prevented puppies from being over-excited around the cat(s), such as controlling and modifying their behaviour with distractions, rewarding calm behaviour, or by punishing puppies for chasing. Whilst there was no evidence in our data that cats or dogs were injured during introductions, it is unclear how puppy chasing behaviour may evolve over time and whether it increases the risk of a dog causing an injury to a cat (or cat injuring a dog) in the future.

In the quantitative analysis, the multivariable logistic regression model revealed that as the number of dogs in the household increased, the odds of ‘only desirable’ behaviours being observed towards household cats also increased. In the UK, the odds of cat ownership are higher in households with no dogs and in households with more than one dog [36]. This suggests that in the UK, cats may be more likely to live in multi-dog households than single-dog households. Potentially having another dog(s) to engage with may have resulted in the puppies showing less interest in the household cat(s). The puppies could have also observed the existing dog(s) and learnt from them how to behave desirably around the cat(s). Fox [11] reported that cats were generally less passive and fearful when interacting with dogs if they had grown-up around them. Therefore, it can be postulated that cats already living in a household with another dog(s) may have been more accustomed to dog behaviour and therefore interacted with the puppy differently compared to a cat that had never lived with a dog before. The qualitative analysis supports this idea: a small number of owners commented that the cat’s previous experience with other dogs meant that they were less concerned about the introduction, sometimes prompting them to rely on pet-led introduction as a result.

One recommendation to aid introducing a puppy to a cat is to ensure the process is gradual and taken slowly over a period of days or weeks, depending on the animals’ reactions. Most participants in this study (40.2%) appeared to be aware of this advice, as typically puppies were introduced ‘gradually (slowly over a period of more than one day)’. The multivariable logistic regression model showed that owners who introduced their puppy ‘quite gradually’ or ‘gradually’ had increased odds of reporting ‘only desirable’ behaviours from their puppy compared to owners who introduced their puppy ‘immediately’ or ‘quite quickly’. Qualitative analysis revealed that owners achieved the gradual introduction by paying attention to the timing and pace, and by manipulating the pets’ environment to ensure the introduction was controlled and supervised. Learning outcomes in dogs are affected by the frequency of reinforcement [37,38]. It could be speculated that a gradual introduction is more likely to be associated with puppies showing ‘only desirable’ behaviour because it gives owners more opportunities to reinforce puppy behaviours that are desirable. A gradual approach to introduction also gives puppies and cats more time to habituate to each other. Habituation (defined as a decrease in intensity or probability of respondent behaviour after regular exposure [39]), could lead to puppies having lower arousal levels around cats and thus showing fewer/less intensive attempts to initiate play or chase. Cats that become habituated to puppies may also run away from puppies less often than unhabituated cats.

Quantitative analysis indicated that the odds of the puppies displaying ‘only desirable’ behaviours were higher when the introduction data were collected when the puppies were less than 12 weeks old compared to 12 to 22 weeks of age. As the ages of the puppies at the time of data collection were calculated using the puppy’s date of birth and the date of survey completion, the duration of time the cat(s) and the puppy had spent together was not measured. However, this finding provides support for the studies by Feuerstein and Terkel [5] and Thompson et al. [10] who reported that the first encounter occurring at an early age was advantageous to establishing an amicable relationship between cohabiting cats and dogs and a better understanding of the other species’ body language.

Two broad approaches to introducing puppies to household cat(s) were identified in the qualitative analysis: owner-led and pet-led. Owners who led on introductions expected cat(s) and dogs to develop amicable behaviours. Owners who opted for pet-led introductions typically expected mutual avoidance, but also accepted behaviours (such as aggression towards dogs, vocalisation, hiding, and changes in general patterns of behaviour) which are typically indicative of signs of stress in cats [40]. Different expectations regarding cat-dog relationships may be due to different personalities of household cat(s). Alternatively, owners’ expectations may be shaped by perceptions of cats’ and dogs’ personalities overall. Compared to cats, dogs are perceived as more playful with the owner and more sociable with other dogs and strangers [41], which may mean that owners interpret avoidance and aggressive behaviour as signs of cats’ independence, influencing their decision to let cats interpose during pet-led introductions. Owners of both cats and dogs have also been reported to see cats as more ‘neurotic’ than dogs [42]. This perception could mean that owners accept cats and dogs living separate lives and regard cat aggression towards dogs as being acceptable rather than context-specific behaviour. Depictions of cat-dog relationships in popular culture may also shape owners’ expectations and consequently their approach to introductions. For instance, the idiom ‘to be/fight like cats and dogs’ has been recorded in over 65 European languages and stories depicting cat-dog conflict have been common in European culture since Medieval times [43]. Education of owners is needed to alter expectations of cat-dog relationships and to dispel this potential preconception that cats and dogs cannot have an amicable relationship. Additionally, as highlighted earlier, there is a need to improve owners’ understanding of signs of stress and body language in cats, and ensure early interactions are monitored.

The cat-dog relationships identified through the qualitative analysis can be imagined on a continuum from friendship, through amicable relationships, avoidance, to conflict. Owners who led on pet introductions reported that over time pets were showing more amicable behaviour, choosing to spend more time in each other’s proximity and in some cases engaging in mutual grooming, co-sleeping and play. This is in line with past research which showed that within a sample of 1270 co-habiting cats and dogs from Brazil, 68.5% of cats and dogs were found to co-sleep and 62.4% engaged in social play [41]. Similar findings were identified by Thomson et al. [10] who identified that 49.2% of dogs and 42.8% of cats were happy to share a bed and 27.1% of pets engaged in daily social play, with a further 12.3% of dogs and 10.3% of cats grooming the other species daily.

A potential limitation of this study is that detailed demographic data about the cats or their behaviour during introductions or daily interactions with puppies were not collected. Information about pets’ personalities, likely to impact on how pets interact and react when introduced [42], were also not collected. Nonetheless, to the authors’ knowledge, this is the first study to explore factors associated with puppies displaying unthreatening behaviour following introduction to existing household cats. This study is unlikely to be influenced by recall bias, as the surveys were issued to owners when their puppies reached specific ages and were only available for the owner to complete for 24 days. In addition, application of a mixed-methods design and confirmation of findings derived through qualitative and quantitative analysis increases the credibility of results. Qualitative analysis additionally helps to expand on the quantitative findings by providing richer context to the associations identified through statistical analysis.

## 5. Conclusions

The study identified that more than a quarter of participants acquiring a puppy already owned at least one cat, nearly four times more than previously reported Murray et al. [1]. Although just 7% of puppies consistently showed unthreatening (‘only desirable’) behaviours towards cats at the time of data collection (age 22 weeks or less), nearly a third of puppies interacted with cat(s) in a calm manner at least some of the time. Very few (1.7%) puppies were reported by their owners to show aggressive behaviour towards cats. However, approximately half of puppies were reported to be over-excited, playful, and chased cats. Two styles of introductions were identified: owner- and pet-led. These styles of introduction were linked with different expectations of cat-dog relationships. Owners who led on introductions hoped for an amicable relationship between cats and puppies, whereas owners who let cats introduce themselves, generally expected puppies and cats to not be friendly with each other. 

Further education of owners around cat-dog interactions is needed to change expectations regarding their relationships and to dispel the myth that cats and dogs cannot have an amicable relationship. Additionally, education around interpretation of body language in cats is recommended, as many participants accepted behaviours from their cat(s) such as aggression and avoidance which are indicative of stress. Improving the understanding of owners is vital to support owners in taking steps aimed at reducing stress in their cat(s), such as monitoring and intervening during cat-dog interactions. The results also suggest that early, gradual, owner-led introductions are important in increasing the chance of positive interactions between puppies and household cats, and should be encouraged when the two species are first introduced.

## Figures and Tables

**Table 1:** Definition and categorization of owner-reported observed puppy behaviour following introduction to existing household cat(s).

Categorisation	Behaviour	Definition Examples
‘Desirable’ behaviour	Interacting in a calm way	Sniffing or sleeping with the cat(s)
	Uninterested or ignored the cat(s)	-
‘Undesirable’ behaviour	Aggressive	Growling or snapping
	Nervous or fearful	Moving into another room or freezing
	Wary or cautious	Keeping a distance or cowering during the interactions
	Chased the cat(s)	-
	Overexuberant or over-excited	Persistently trying to interact
	Played with the cat(s)	-

**Table 2:** List of responses available to owners to describe the speed of introducing their puppy to existing household cat(s).

Introduction Speed	
Immediately (they were together straight away)	
Quite quickly (interaction was controlled for up to the first 2 h after they met)	
Quite gradually (their meeting was gradual over the first day)	
Gradually (they were introduced slowly over a period of more than one day)	487 (40.2)

**Table 3:** Owner-reported introduction speed of 1211 puppies to existing household cat(s).

Introduction Speed	Number (%)
Immediately (they were together straight away)	243 (20.1)
Quite quickly (interaction was controlled for up to the first 2 h after they met)	229 (18.9)
Quite gradually (their meeting was gradual over the first day)	252 (20.8)
Gradually (they were introduced slowly over a period of more than one day)	487 (40.2)

**Table 4:** Univariable logistic regression models of odds ratios (ORs), 95% confidence intervals (CIs) and *p* values of potential explanatory variables for ‘only desirable’ behaviour from puppies following introduction to existing household cat(s).

Variable	Category	‘Only Desirable’ Behaviours *n* (%)	‘One or More Undesirable’ Behaviours *n* (%)	OR (95% CI)	*p* Value
Sex of puppy	Male	50 (8.1)	569 (91.9)	1.00	
	Female	38 (6.4)	554 (93.6)	0.78 (0.50–1.21)	0.267
Number of cats in household	Continuous variable			1.05 (0.92–1.19)	0.482
Number of dogs in household	Continuous variable			1.28 (1.14–1.43)	<0.001
Another dog aged ≥ 1 year in household	No	33 (4.6)	690 (95.4)	1.00	<0.001
	Yes	55 (11.3%)	433 (88.7)	2.66 (1.70–4.16)	
Introduction speed ^1^	Immediately/quite quickly	24 (5.1)	448 (94.9)	1.00	
	Gradually/quite gradually	64 (8.7)	675 (91.3)	1.77 (1.09–2.87)	0.021
Age puppy joined household	Continuous variable			1.00 (0.99–1.02)	0.596
Age of puppy when cat-dog data were collected	12 to 22 weeks	55 (5.5)	951 (94.5)	1.00	<0.001
	<12 weeks	33 (16.9)	162 (83.1)	0.28 (0.18–0.45)	
Kennel Club group ^2^	Non purebred dog and all other Kennel Club groups	69 (6.6)	982 (93.4)	1.00	0.018
	Pastoral	19 (11.9)	141 (88.1)	1.92 (1.12–3.28)	

^1^ ‘Immediately’ defined as ‘they were together straight away’, ‘Quite quickly’ defined as ‘interaction was controlled for up to the first 2 h after they met’, ‘Quite gradually’ defined as ‘their meeting was gradual over the first day’, and ‘Gradually’ defined as ‘they were introduced slowly over a period of more than one day’. ^2^ The ‘Non purebred dog and all other Kennel Club groups’ category included all dogs reported by their owners to not be a pedigree dog or within the following Kennel club groups: Gundog, Hound, Terrier, Toy, Utility and Working.

**Table 5:** Multivariable logistic regression model for ‘only desirable’ behaviour from puppies following introduction to existing household cat(s) (*n* = 1201).

Variable	Category	‘Only Desirable’ Behaviours *n* (%)	‘One or More Undesirable’ Behaviours *n* (%)	OR (95% CI)	*p* Value
Number of dogs in household	Continuous variable			1.31 (1.16–1.46)	<0.001
Introduction speed ^1^	Immediately/quite quickly	24 (5.1)	448 (94.9)	1.00	0.013
	Gradually/quite gradually	64 (8.7)	675 (91.3)	1.86 (1.14–3.05)	
Age of puppy when cat-dog data were collected	12 to 22 weeks	35 (4.9)	681 (95.1)	1.00	<0.001
	<12 weeks	53 (10.7)	442 (89.3)	2.52 (1.60–3.96)	

^1^ ‘Immediately’ defined as ‘they were together straight away’, ‘Quite quickly’ defined as ‘interaction was controlled for up to the first 2 h after they met’, ‘Quite gradually’ defined as ‘their meeting was gradual over the first day’, and ‘Gradually’ defined as ‘they were introduced slowly over a period of more than one day’.

**Table 6:** Themes, sub-themes and quotes that illustrate cat-dog relationships and owners’ approaches to, and expectations of, introducing a puppy to a household cat(s). Text in bold highlights the crucial content.

Theme	Sub-Theme	Quotes
1. Owner-led introduction	Pace and timing	“We let them play **for short intervals** and stop before they get over excited/hurt” “I **gradually** introduce the puppies to our cats from **around 4 weeks of age (when I feel comfortable that the cats will not harm them)**”
	Manipulating pets’ physical environment	“Prior to getting [the puppy], **we set our house up with stairgates and see-through room dividers**” “We have her food/water bowls upstairs **in a safe space, so she doesn’t have to be around the puppy** in order to eat or drink” “They [the cats] have **plenty of spaces high up to get away from her** [the dog] (…)”
	Controlling and modifying pet behaviour	“We have not allowed [the puppy] to get too excited around them [the cats], **and we calmly divert her attention** if her arousal levels become too high” “When [the puppy] has spotted her [the cat] we have **rewarded calm behaviour**” “[The puppy] is not allowed to be loose with the cats, **she is always on a training line**” “We **scold her [the puppy] verbally each time she does it** [chases cats] and sometimes **remove her from the area** to give them [the cats] an opportunity to go wherever they want to without fleeing for their lives” “Today she [the cat] came most of the way down whilst he [the puppy] was at the foot of the stairs and I **drip fed them both high value food (chicken) to reinforce the positive, calm interaction**”
	Supervision	“At the moment **we are monitoring their interaction and don’t leave them alone in the same room**” “Introductions between [the puppy] and the cats have been **closely supervised**, on-lead or through the stairgates or room dividers”
2. Pet-led introduction	Allowing cat(s) to intervene	“**They [the cats] introduced themselves.** They are well able to look after themselves if they choose to get on the floor” “Cats **put him in his place,** and they play now, and the **cats are the boss**” “One cat is **slowly sorting the puppy out**” “If [the puppy] ever pushes it too far and the cat doesn’t want to play anymore **the cat will give her a bop on the nose** (without her being hurt)”
	Cat(s) can choose	“Their introduction will go **as quickly as the cat wants**” “The cats have their own access to the house away from [the puppy] **so they can adapt to a boisterous puppy** in the house” “We have kept [the puppy] apart from the cats where possible, **allowing the cats to choose when to come near**”
3. Emerging cat-dog relationship	Friendship	“There’s a little **bit of grooming each other**. And they often **fall asleep next to each other**” “Often it is the **cat that starts the play fighting**. At other times **they play very gently or play jointly with a toy**, sometimes **they sleep together**”
	Amicable relationship	“She [the puppy] has been **relatively calm** with the cats so they have increasingly **tolerated being near her**” “After 1 week **cat chose to sit in lounge where dog was**” “She [the cat] **has come and sniffed [the puppy],** and walked away” “They are **co-existing** well”
	Mismatched needs and misunderstandings	“[The puppy] **is really curious** about the cats, he will stand still and **watch them** then slowly approach, **but they run away**” “[The puppy] is very inquisitive and wants to play with everyone and everything, **he is learning slowly that cats don’t play**” “He [the puppy] then **chases them** because they run, **so he thinks it’s a game and he wants to play**”
	Separate lives	“The girl cat (…) does not like the dogs **so she stays away or keeps her distance**” “Mostly they are calm and **tend to ignore each other**”
	Conflict and fear	“[The cat] is **not impressed as all**. In fact, I think he’s a **little frightened of her**” “They will all sit together for treat but [the puppy] is cautious as she [the cat] is a cat that **ALWAYS growls**” “**[The cat] hits [the puppy] a lot** but rarely with claws out (…) [the cat] **often runs away from [the puppy]**”
4. Expectations about cat-dog relationship	Expecting tolerance	“They [the cats] are not anxious or aggressive towards him [the puppy], they **just tolerate him and stay out of his way**” “One cat treats [the puppy] with **gentle disdain** and they are **mutually tolerant** to the point where [the puppy] allows the cat to share his food”
	Expecting respect	“[The cat] has been wary of [the puppy] but she will correct his behaviour which I think is good for him, **so he learns to be respectful as early as possible**” “[The cat] is a lot tougher and **has shown her [the puppy] who’s boss and [the puppy] respects that** it seems and doesn’t chase said cat, but is still dead keen to be mates” “The cat took one look at me “what have you done!” **[The puppy] is respectful of cat now**”
5. Context of introductions	Layout of the house	“We had to introduce them quickly as it **would have been too difficult to keep separate due to house layout**”
	Cat’s history	“The **cat is a rescue** (…) and has socialisation issues from her previous experiences and is unlikely to tolerate a formal introduction” “[The cat] **has been living with my previous dog** since we rescued when she was 4 years old. [The cat] is a friendly (…) however **she is now 12 years old** and [the puppy] is very bouncy around her”
	Dog’s history	“[The puppy] is complete happy around the cats (…) we think this is because she was **raised with cats in the breeder’s household**”

## Data Availability

The data are not publicly available due to ethical approval of participant informed consent that included Generation Pup participants being informed that we will remove all personally identifiable information before sharing data with Universities and/or research institutions.
